# Red Blood Cell-Based Delivery Systems for the Release of Hemoglobin-Derived Peptides with In Vitro Antitumor Activities

**DOI:** 10.3390/ph18040570

**Published:** 2025-04-14

**Authors:** Cínthia Caetano Bonatto, Graziella Anselmo Joanitti, Luciano Paulino Silva

**Affiliations:** 1 Postgraduate Program in Animal Biology, University of Brasilia (UnB), Brasília 70910-900, DF, Brazil; cinthiabonatto@gmail.com; 2 Laboratory of Bioactive Compounds and Nanobiotechnology (LCBNano), Faculty of Health Sciences and Technologies, University of Brasilia, Metropolitan Center, Ceilandia 72220-275, DF, Brazil; gjoanitti@unb.br; 3 Embrapa Genetic Resources and Biotechnology, Nanobiotechnology Laboratory (LNANO), Parque Estacao Biologica, Final W5 Norte, Brasília 70770-917, DF, Brazil

**Keywords:** liposome, red blood cell, antitumor, in vitro, cell mimetics, peptides, nanosystems, mammalian cell

## Abstract

**Background/Objectives:** This study aimed to develop liposomes derived from lipids obtained from red blood cell membranes for potential use in antitumor applications. Hemoglobin hydrolysates exhibiting peptides with known antitumor activities were encapsulated within these liposomes. **Methods:** The developed liposomal systems were characterized by their physicochemical properties, including size, surface charge, and encapsulation efficiency, and tested in vitro against 4T1 breast cancer cells and NIH3T3 fibroblasts. **Results:** Results indicated that the liposomes achieved effective encapsulation (88.9%), with nanometer-scale sizes (ranging from 140.7 nm for Blank-Liposomes to 658.3 nm for Pep-Liposomes) and stable colloidal properties. **Conclusions**: Although cytotoxicity was limited, the use of liposomes from endogenous components, such as red blood cells, demonstrates promise as a complementary approach in anticancer therapy.

## 1. Introduction

Blood cells, also known as erythrocytes or red blood cells, are vital components of blood tissue. Studies involving erythrocytes have been conducted in various fields of knowledge, including biology, medicine, and veterinary medicine. Consequently, the extensive literature describes the characteristic shapes and dimensions of red blood cells across different species [1,2]. In most mammals, mature circulating erythrocytes are anucleated biconcave disks with varying volumes and average diameters. Their primary function is to transport oxygen (O_2_) from the lungs to the tissues and carbon dioxide (CO_2_) from tissues back to the lungs, making them a highly specialized transport system [3].

The study of erythrocyte physiology and structural characteristics has opened new avenues for their application as components and bioactive agents in delivery systems. This interest arises from their capacity to circulate throughout the body and to direct therapeutic agents to specific sites, including the reticuloendothelial system. Erythrocytes offer advantages such as biocompatibility, reduced toxicity, low immunogenicity, zero-order release kinetics (i.e., gradual drug release), and biodegradability through normal metabolic pathways in the spleen and liver. Additionally, they offer ease in preparation due to their simple formulation and high drug encapsulation potential in small volumes [4,5,6].

Cancer remains a critical global health challenge, annually affecting millions worldwide. Current treatments, including surgery, chemotherapy, and radiotherapy, often result in severe side effects due to their non-specific action on both healthy and cancerous cells, which highlights the need for novel therapeutic agents and more efficient drug delivery systems. In this context, nanobiotechnology has emerged as a promising field, providing advanced drug delivery systems options able to have sustained-release mechanisms that minimize damage to healthy tissues while effectively targeting tumor cells [7].

Among the innovative approaches in this field is the use of red blood cell (RBC) mimetics [8]. RBCs, which are naturally biocompatible and abundant, have been explored for their ability to deliver bioactive compounds systemically. Hemoglobin, the primary RBC component, can be hydrolyzed to produce peptides with known bioactivities, including anticancer properties [9]. By leveraging RBC structures, it is possible to design and create delivery systems that combine the bioactive properties of hemoglobin-derived peptides with RBCs’ natural delivery capabilities [8].

In this study, we developed RBC-based nanosystems for delivering antitumor hemoglobin-derived peptides and evaluated their effects on a breast cancer cell line (4T1) in vitro. The nanostructures were characterized using various techniques, including measurements of the hydrodynamic diameter, polydispersity index (PdI), Zeta potential, and encapsulation efficiency (assessed via UV-Vis spectrophotometry). Finally, we evaluated the cytotoxicity of the nanosystems in a non-tumoral mammalian cell line (NIH3T3) in vitro.

## 2. Results and Discussion

### 2.1. Obtaining Hemoglobin-Derived Peptides

**Figure 1:**
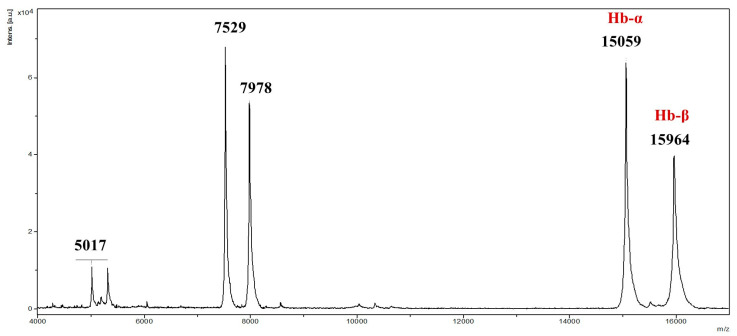
MALDI-TOF mass spectrum of bovine hemoglobin extract acquired in linear positive mode at m/z range of 2000–20,000, showing monomers of α and β chain ions in their singly charged ([M+H]+ = 15,059/15,964) and doubly charged ([M+2H]+ = 7529/7978) forms.

**Figure 2:**
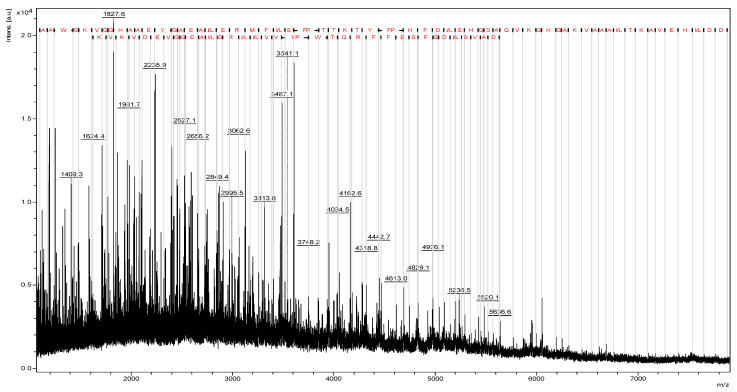
Mass spectrum of bovine hemoglobin obtained by MALDI-TOF mass spectrometry in ISD mode, depicting α (top) and β (bottom) chains.

To ensure structural integrity, commercial non-hydrolyzed bovine hemoglobin underwent MALDI-TOF mass spectrometry analysis, confirming ions with *m*/*z* ranging from 2000 to 20,000, consistent with intact α and β chains (Figure 1). Prior to hydrolysis, MALDI-TOF mass spectrometry confirmed the integrity of the bovine hemoglobin, with no detectable degradation products. This ensured that the subsequent peptide obtaining process originated from intact hemoglobin. In the source fragmentation of the extracted total hemoglobin, sequences of 66 and 36 amino acid residues were revealed, correlating with α and β chains, respectively (Figure 2). Studies indicate that hemoglobin hydrolysis produces bioactive peptides [9,10,11,12,13]. According to Ivanov et al. (2005), Hemoglobin-derived peptides arise from enzymatic hydrolysis or pH alterations [10].

**Figure 3:**
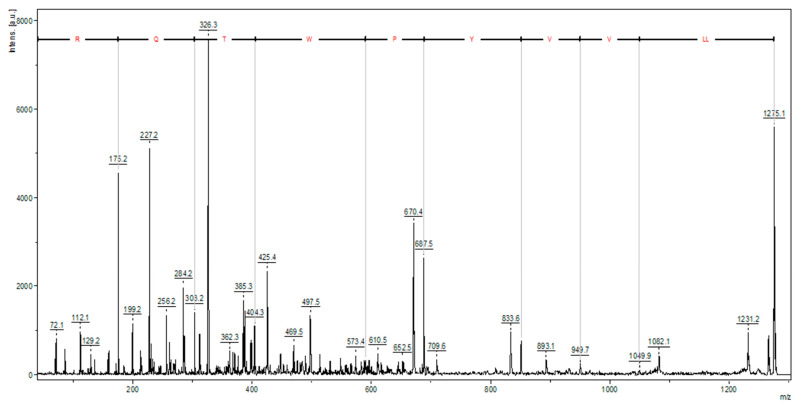
Mass spectrum obtained by MALDI-TOF/TOF operated in LIFT^TM^ mode of precursor ion at [M + H]+ = 1275.1 (identified as hemorphin), with y-series ions labeled, derived from bovine hemoglobin tryptic hydrolysate.

The trypsin hydrolysis of bovine hemoglobin yielded a high-intensity ion, identified as a hemorphin (LLVVYPWTQR) (Figure 3). Hemorphins are bioactive opioid peptides derived from hemoglobin β chain proteolysis, often containing a Tyr-Pro-Trp (YPW) motif and various N- and/or C-terminal modifications. Hemorphin-3 to -7 have truncations in the C-terminal regions. Hemorphin subfamilies like V-hemorphins, VV-hemorphins, and LVV-hemorphins [14,15] display diverse biological effects, including antitumor activity [16,17].

### 2.2. Cytotoxicity of Intact Hemoglobin and Its Derived Peptides in Tumoral and Non-Tumoral Cell Lines In Vitro

**Figure 4:**
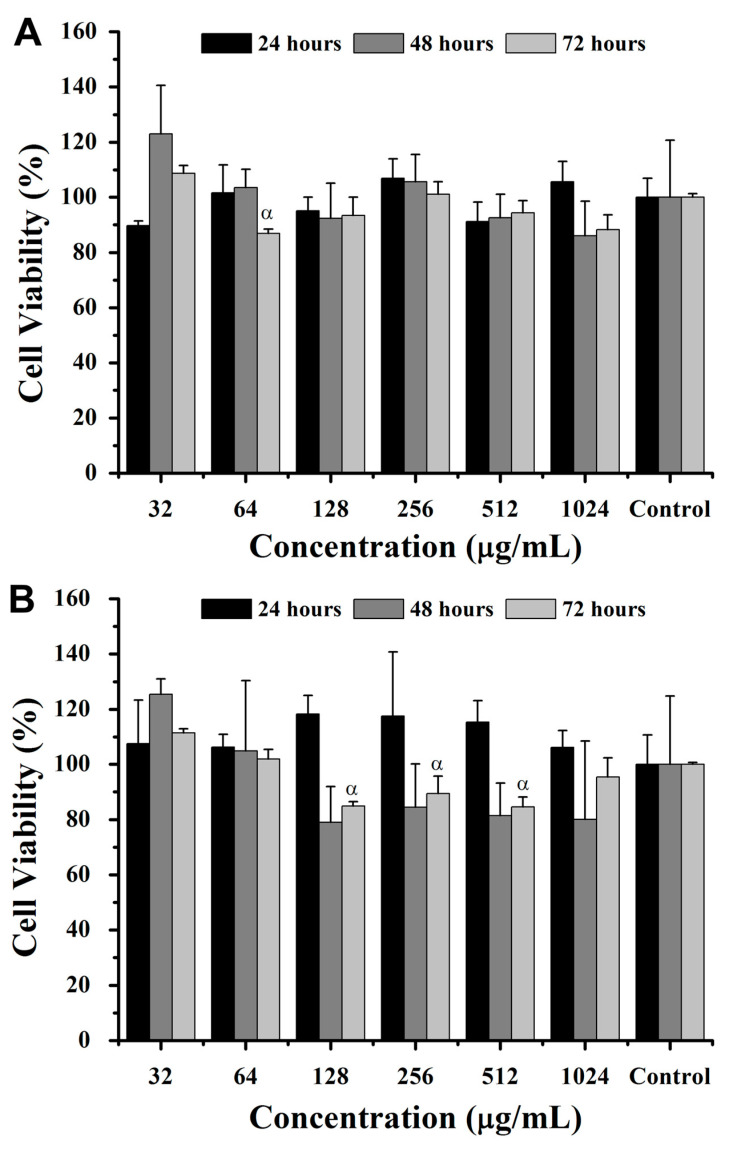
Viability of breast cancer cells (4T1, **A**) and non-tumoral murine fibroblasts (NIH/3T3, **B**) (MTT assay) after 24, 48, and 72 h of exposure to intact bovine hemoglobin. ANOVA: significant difference among groups, *p* < 0.05 (Tukey post hoc test). Statistically significant difference (*p* < 0.05) relative to 32 μg/mL (α).

**Figure 5:**
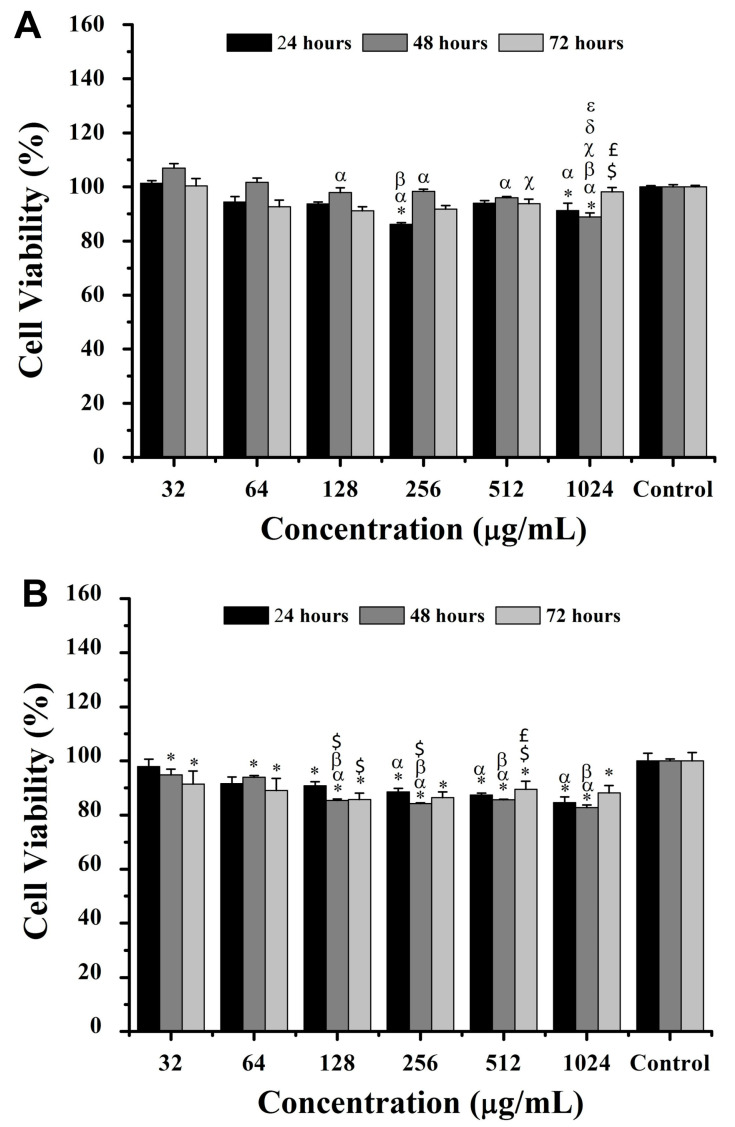
Viability of breast cancer cells (4T1, **A**) and murine fibroblasts (NIH3T3, **B**) (MTT assay) after 24, 48, and 72 h of exposure to peptides resulting from hydrolysis of bovine hemoglobin by trypsin. ANOVA: significant difference among groups, *p* < 0.05 (Tukey post hoc test). Statistically significant difference (*p* < 0.05) relative to control: 0 μg/mL (*), 32 μg/mL (α), 64 μg/mL (β), 128 μg/mL (χ), 256 μg/mL (δ), 512 μg/mL (ε), 24 h ($), and 48 h (£).

The incubation of intact hemoglobin and its derived peptides in breast cancer cells (4T1) and fibroblast cells (NIH/3T3) was performed for 24, 48, and 72 h at concentrations of 32, 64, 128, 256, 512, and 1024 μg/mL (Figure 4 and Figure 5). The results showed native hemoglobin’s limited impact on 4T1 cell viability, with a significant reduction of ~20% at 64 μg/mL after 72 h when compared to 32 μg/mL (*p* < 0.05) (Figure 4A). Similarly, NIH3T3 cells exhibited reductions of ~8%, ~24%, and ~24% in cell viability at 128, 256, and 512 μg/mL, respectively, after 72 h compared to 32 μg/mL (Figure 4B). Despite these specific differences, there were no significant variations in cell viability at any concentration or time point when compared to the control without intact hemoglobin incubation.

For hemoglobin-derived peptides, after 24 h, 4T1 cell viability decreased significantly (*p* < 0.05) by ~7% and ~9% at 256 and 1024 μg/mL, respectively, compared to the control (Figure 5). Similarly, NIH/3T3 cells exhibited a viability decrease of ~15%, ~13%, and ~11% at 256, 512, and 1024 μg/mL, respectively. At 48 h, 4T1 cell viability decreased by ~11% at 1024 μg/mL compared to all other concentrations, while NIH/3T3 cells showed up to a ~17% viability reduction at concentrations above 128 μg/mL (Figure 5). At 72 h, 4T1 cell viability increased by ~7% at 512 μg/mL versus 128 μg/mL, whereas all concentrations reduced NIH/3T3 viability compared to the control (*p* < 0.05) (Figure 5).

Once the release of proteases with trypsin-like activity by cells in culture was reported [18,19], intact hemoglobin was applied to both cell lines over various time points. The evaluation of the results indicated a marked decrease in the viability of 4T1 cells, suggesting the continuous and progressive formation of peptides derived from hemoglobin with varying sizes, which would be directly related to their activity to varying degrees [20]. Aiming to prevent in vitro peptide degradation, particularly of hemorphin, into smaller fragments, by proteases secreted by cells, hemoglobin hydrolysate with a protease inhibitor cocktail was used for the treatment of the two cell lines at different times. The results indicated a dose-dependent decrease in the viability of 4T1 cells of up to 20% and up to 22% for NIH3T3 cells after 48 h, suggesting the possible inhibition of the proteolytic degradation of peptides in the long term.

Given the low rates of inhibition of cell viability promoted by the hemoglobin-derived peptides in the breast cancer (4T1) and fibroblasts (NIH/3T3) cells, we opted to encapsulate these peptides to enhance their activity solely against 4T1 cells. In this context, we formulated liposomes from lipids derived from red blood cell membranes.

### 2.3. Physicochemical Characteristics of Liposomes

**Figure 6:**
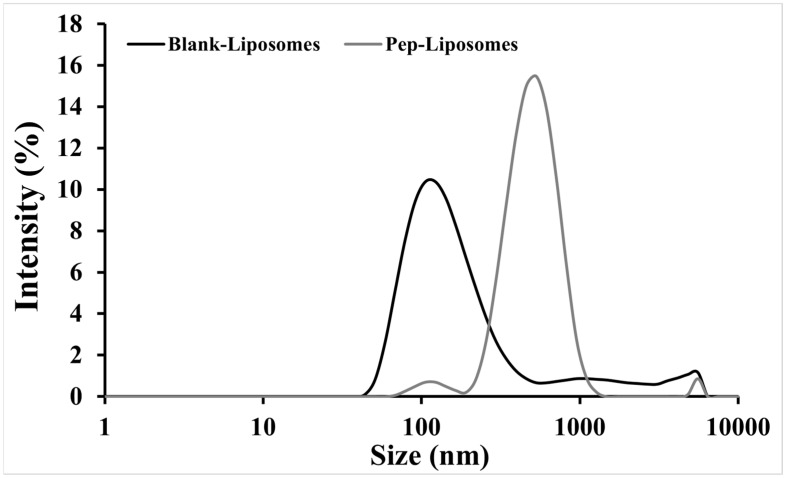
The size distribution (hydrodynamic diameter—Dh) profiles of Blank-Liposomes and Pep-Liposomes. The larger size of Pep-Liposomes correlates with hemoglobin-derived peptides’ encapsulation, as described in Table 1. Measurements were performed at 25 °C using three independent replicates.

Dynamic light scattering evaluation revealed variations in size, the polydispersity index (PdI), and the Zeta potential (ZP) of Blank-Liposomes and Pep-Liposomes (Table 1). There was an increase (516.7 nm) in the hydrodynamic diameter (Dh) and PdI and a decrease in the Zeta potential (+5.7 mV) after the encapsulation of hemoglobin-derived peptides when compared to the Blank-Liposomes (Table 1), which exhibited an encapsulation efficiency of ~89%. In addition, Figure 6 demonstrates the distinct size distributions of Blank-Liposomes and Pep-Liposomes. The increase in Pep-Liposomes’ size distribution reflected the successful encapsulation of hemoglobin-derived peptides, which altered lipid packing and increased vesicle dimensions.

Liposomes have been used for the encapsulation of therapeutic proteins and other molecules, a well-established fact in the literature. They can be used both for controlled drug release and the delivery of their contents directly into the cytoplasm of cells [21,22]. We investigated the potential of formulating liposomes using lipids extracted from red blood cell membranes and evaluated their efficiency in encapsulating hemoglobin-derived peptides. The liposomes displayed increased Dh when formulated in the presence of peptide hydrolysate, as well as an increased PdI and reduced Zeta potential. Studies have shown that liposomes produced from synthetic phospholipids (single-molecular components) result in uniform systems with unimodal distributions in the PdI [23,24]. In contrast, liposomes developed from complex components tend to form more heterogeneous systems with polymodal distributions in PdI. The Blank-Liposomes showed a lower Zeta potential compared to Pep-Liposomes, suggesting the presence of cationic peptides or other biomolecules exposed on the surface.

The Zeta potential is considered a key parameter for evaluating the colloidal stability of liposomes, representing the surface charge of nanostructures and their electrostatic repulsion in suspensions. Values between −30 mV and +30 mV generally indicate moderate colloidal stability, where aggregation/agglomeration is minimized but not entirely prevented. In this study, the Pep-Liposomes exhibited a Zeta potential of −6.4 ± 0.7 mV, suggesting incipient colloidal stability. This low stability may have been influenced by the surface-exposed peptides from the hemoglobin hydrolysate, which could partially neutralize the liposomal surface charge. Despite this, the system remained sufficiently stable for the experimental conditions evaluated.

### 2.4. Liposome Cytotoxicity

**Figure 7:**
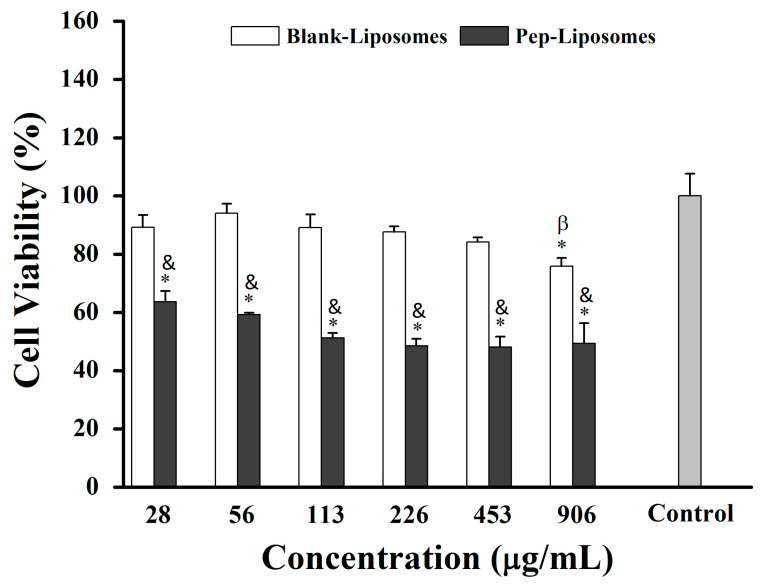
Evaluation of viability of 4T1 breast cancer cells by the MTT assay after 24 h of exposure to Blank-Liposomes and Pep-Liposomes (loaded with hemoglobin-derived peptides). Statistically significant difference (*p* < 0.05) relative to control: 0 μg/mL (*), 56 μg/mL (β), and Blank-Liposomes (&). ANOVA: significant difference among groups, *p* < 0.05 (Tukey post hoc test). Different symbols indicate statistically significant differences among groups.

Liposomes have been used to encapsulate therapeutic proteins and other drugs, as is well established in the literature. They can be used both for controlled drug release and for delivering their contents directly into the cell cytoplasm [22,25]. In this context, we investigated the formulation of liposomes using lipids extracted from red blood cell membranes and their potential to encapsulate hemoglobin-derived peptides. All concentrations of Pep-Liposomes (with hemoglobin-derived peptides) significantly reduced 4T1 breast cancer cell viability by 37% to 51% compared to the control after 24 h of incubation, although a concentration-dependent effect was not observed (Figure 7, *p* < 0.05). At all concentrations, Pep-Liposomes decreased cell viability when compared to Blank-Liposomes (Figure 7, *p* < 0.05). The Blank-Liposomes were active at the highest concentration, reducing the viability, indicating cytotoxicity at the lipid concentration used. Some phospholipids in liposome formulations and membrane constituents, when oxidized, can induce apoptosis depending on chemical structure, cell concentration, and cell type [26]. The cytotoxicity of Pep-Liposomes may indicate a synergistic effect between hemoglobin-derived peptides and liposome lipids [27], suggesting that heme groups participate in lipid oxidation, implying that the heme group in hydrolysate may lead to the oxidation of liposome membranes or tumor cells [25]. FRUHWIRTH et al. (2008) reported that the oxidation of some membrane phospholipids induces apoptosis depending on their chemical structure, cell concentration, and cell type [25]. Future studies are needed to clarify these aspects.

Another potential factor influencing cytotoxicity could be the concentration of lipids in the liposome formulation. High lipid concentrations can exhibit cytotoxic effects on certain cell types, potentially affecting cellular viability independently of peptide presence [28]. This effect may stem from lipid aggregation or membrane disruption mechanisms, which become more pronounced at elevated lipid levels [29]. Such effects have been particularly noted in formulations with excessive lipid content, where cellular stress responses, including apoptosis, may be triggered. Further investigation is required to determine whether the lipid concentration of Blank-Liposomes contributes to their observed cytotoxicity in specific cell types such as in 4T1 cells.

Leveraging the innate biocompatibility and natural targeting potential of red blood cells, this innovative delivery system holds a significant promise for mitigating the undesired side effects commonly seen in conventional therapies. The tunable nature of these systems, coupled with their robust potential for functionalization, creates new opportunities for integrating targeting ligands. This customization could substantially increase specificity for cancerous tissues and organs, enhancing therapeutic precision. Such advancements represent a stride toward therapies that not only maximize efficacy but also minimize collateral impact on healthy cells, aligning with the evolving goals of targeted and patient-centered oncology treatments.

## 3. Materials and Methods

### 3.1. Extraction of Peptides Derived from Hemoglobin

Commercial bovine hemoglobin (Merck, Darmstadt, Germany) underwent enzymatic hydrolysis to generate peptides with potential antitumor activity, using immobilized trypsin (Thermo Scientific, Rockford, IL, USA). Initially, 10 μL of the immobilized trypsin was added to 200 μL of 100 mM ammonium bicarbonate buffer (pH 8.2) following the manufacturer’s instructions. The enzyme solution was then centrifuged, and the supernatant was discarded to remove any enzyme inhibitors. This washing procedure was repeated three times. Afterward, 10 μL of the washed immobilized enzyme was added to 90 μL of the hemoglobin aliquot in 90 μL of 50 mM ammonium bicarbonate buffer (pH 8.2) and incubated for 72 h at 37 °C. After incubation, the samples were centrifuged at 120 g for 1 min, and the enzyme-free supernatant was collected and stored at −80 °C.

### 3.2. Mass Spectrometry Analysis

Aliquots of the intact and hydrolyzed hemoglobin samples were co-crystallized with a saturated matrix solution of alpha-cyano-4-hydroxycinnamic acid (Bruker Daltonics, Bremen, Germany) (1:3, *v*/*v*) or 1,5-diaminonaphthalene (DAN) (Bruker Daltonics, Bremen, Germany), onto a MSP 96-type plate (53 × 41 mm). After drying at room temperature, molecular masses were measured using a MALDI-TOF MicroFlex mass spectrometer (Bruker Daltonics, Germany) with external calibration in positive linear mode over the *m*/*z* range 2000–20,000. Further analysis was conducted using a MALDI-TOF/TOF UltraFlex III mass spectrometer (Bruker Daltonics, Bremen, Germany) with external calibration in positive reflector mode over the *m*/*z* ranges 100–1000 and 600–4000, with MS/MS in LIFT^TM^ mode for precursor ion fragmentation and ISD (in-source decay) to a fragmentation process that occurred in the ion source.

### 3.3. Lipid Extraction from RBCs and Liposome Production

The lipids used for liposome production were derived from female Balb/c mice (2–3 months). The animals were housed in controlled conditions (12 h light/dark cycle, 23 °C, 55% humidity) at the Institute of Biological Sciences, University of Brasilia and had ad libitum access to water and feed. Anesthesia was formulated with 70 μL of 10% ketamine (Dopalen) (Syntech, Bend, OR, USA) and 40 μL of 2% xylazine (Anasedan) (Bayer, Shawnee MI, USA), and peripheral blood was drawn via cardiac puncture. Collected blood (1 mL) was stored in tubes with 0.1% ethylenediamine tetraacetic acid (EDTA) as an anticoagulant and kept at 4 °C for up to 24 h. The study protocol was approved by the Animal Use Ethics Committee (CEUA) of the Institute of Biological Sciences at the University of Brasília (UnBDOC no. 131758/2012).

As a next step, one milliliter of the collected blood was centrifuged at 120× *g* for 10 min at 4 °C, with approximately 400 μL of serum removed. The pellet was washed with 100 mM sodium phosphate buffer (PBS) (Laborclin, Pinhais, Brazil) and centrifuged twice more, yielding a final volume of approximately 800 μL. The cells were then frozen in liquid nitrogen and lyophilized to obtain a red blood powder which was stored at 4 °C until use. For the preparation of liposomes, red blood membrane powder (22.5 mg) was weighed and resuspended in 500 μL of ultrapure water to form a homogeneous solution. Then, 625 μL of chloroform (J.T.Baker, Phillipsburg, NJ, USA), 1200 μL of methanol (J.T.Barker, Phillipsburg, USA), 625 μL of chloroform (J.T.Baker, Phillipsburg, USA), and 625 μL of ultrapure water were sequentially added to each solvent, followed by vortexing for 5 min. After a 3 min centrifugation at 8000× *g* at RT, the chloroform (organic) phase containing lipids was separated and subjected to rotaevaporation at 40 °C for 1 h under 207 Pa to form a lipid film. The film was resuspended with 1.5 mL of 0.5% saline (Laborclin, Pinhais, Brazil), containing hemoglobin-derived peptides (3.26 μg/mL) and vortexed for 5 min. Vesicle size was reduced via extrusion through 100 nm polycarbonate membranes.

### 3.4. Encapsulation Efficiency

Encapsulation efficiency was measured by separating non-encapsulated hemoglobin-derived peptides. Liposomes, with or without peptides, were placed on Amicon Ultra filters (100 kDa exclusion limit) (Merck, Darmstadt, Germany) and centrifuged at 13,000× *g* for 10 min at 4 °C. Each cycle included a 250 μL 0.5% saline wash to remove free hemoglobin-derived peptides. The upper and lower filter fractions were stored at 4 °C and −80 °C, respectively. The absorbance was measured at 280 nm using an espectrophotometer, and the concentration was calculated from a previously established absorbance calibration curve (R^2^ = 0.99).

### 3.5. Dynamic Light Scattering and Zeta Potential

The hydrodynamic diameter (Dh), polydispersity index (PdI), and Zeta potential of liposomes were measured immediately after liposome preparation (time zero) using dynamic light scattering (DLS) and electrophoretic mobility, respectively, using a ZetaSizer Nano ZS, with He-Ne laser (4 mW) at 633 nm. Three measurements were performed at room temperature (25 °C) with a pH of 7.0 and light scattering detected at 173°. The Dh was reported as Z-Average ± standard deviation, whereas the PdI reflected the distribution width within individual measurements.

### 3.6. Cell Viability Assay

Murine 4T1 breast cancer cells and non-tumoral NIH/3T3 fibroblast cells were cultured in Dulbecco’s Modified Eagle Medium (DMEM) (supplemented with 10% fetal bovine serum (Gibco, Grand Island, NE, USA)) and 1% penicillin–streptomycin (Invitrogen Thermo Fisher Scientific, Grand Island, NE, USA). Cells (5 × 10^3^ per well) were seeded in 96-well plates and incubated at 37 °C and 5% CO_2_ for 24 h. Each well received 50 μL of free hemoglobin at different concentrations (32 to 1024 µ/mL), hemoglobin-derived peptides, or liposomes (with or without hemoglobin-derived peptides). After 24, 48, or 72 h, media was removed, and 150 μL MTT solution (15 μL of 5 mg/mL MTT (3(4,5-dimethylthiazol-2-yl)-2,5-diphenyltetrazolium bromide (Invitrogen, Carlsbad, CA, USA) in 135 μL DMEM) was added to each well and incubated for 2 h at 37 °C. Afterwards, MTT solution was replaced with 200 μL dimethyl sulfoxide (DMSO) (Sigma-Aldrich Co., St. Louis, IL, USA) to solubilize formazan, with absorbance measured at 595 nm on a spectrophotometer coupled to a microplate reader (SpectraMax, Molecular Devices, San José, CA, USA). The results were expressed relative to untreated cells (normalized to 100% viability), which served as the negative control; thus, the explicit inclusion of the negative control bar was omitted to streamline data presentation while maintaining comparative clarity.

### 3.7. Statistical Analysis

Biological results were presented as the mean ± standard error of the mean (SEM). Statistical significance was determined using analysis of variance (ANOVA) with Tukey’s test with PAST software (version 2.17b) [30], setting the significance level at *p* < 0.05.

## 4. Conclusions

This study demonstrates the potential of hemoglobin-derived peptides encapsulated within RBC-based nanosystems as targeted anticancer agents. The successful extraction, encapsulation, and characterization of hemoglobin-derived peptides confirm their stability and in vitro antitumor activity, particularly with enhanced efficacy observed in liposomal formulation against a mammalian breast cancer cell line (4T1). By harnessing the inherent biocompatibility and targeting capabilities of red blood cells, this innovative delivery system shows promise in reducing undesired side effects associated with conventional therapies. Their tunability and capacity for functionalization open the door for the integration of targeting ligands, which could increase their specificity for cancerous tissues. Future work will focus on in vivo assessments and optimizing encapsulation efficiency to further validate the therapeutic potential of these RBC nanosystems in targeted cancer treatment.

## 5. Patents

Patent Number: WO/2016/119030: Method for Obtaining Bioactive Molecules in Microstructured and Nanostructured Carrier Systems. Inventors: Luciano Paulino da Silva, Cinthia Caetano Bonatto, and Graziella Anselmo Joanitti. Assignees: Embrapa—Empresa Brasileira de Pesquisa Agropecuária and Fundação Universidade de Brasília (FUB) [BR]. International Filing Date: 15 January 2016. International Application Number: PCT/BR2016/050001. Publication Date: 4 August 2016. Priority Data: BR102015002069-4, 29 January 2015 (Brazil).

## Figures and Tables

**Table 1:** Physicochemical characteristics of liposomes and liposomes with peptides.

	Dh (nm)	PdI	ZP (mV)	E.E. (%)
Blank-Liposomes	140.7 ± 3.3	0.328 ± 0.010	−12.1 ± 1.0	-
Pep-Liposomes	658.3 ± 42.2	0.500 ± 0.061	−6.4 ± 0.7	88.9

Dh (hydrodynamic diameter), polydispersity index (PdI), Zeta potential (ZP), and encapsulation efficiency (E.E.). Values represent means of three independent readings.

## Data Availability

The original contributions presented in this study are included in the article. Further inquiries can be directed to the corresponding author.

## Abbreviations

Dh	Hydrodynamic diameter
PdI	Polydispersity index
ZP	Zeta potential
DAN	1,5-diaminonaphthalene
PBS	Phosphate buffer
DMEM	Dulbecco’s Modified Eagle Medium
MTT	3(4,5-dimethylthiazol-2-yl)-2,5-diphenyltetrazolium bromide
DMSO	Dimethyl sulfoxide
